# iPSC-derived exosomes promote angiogenesis in naturally aged mice

**DOI:** 10.18632/aging.204845

**Published:** 2023-06-26

**Authors:** Xingyu Li, Heng Zhang, Xuemeng Wang, Meng Lu, Qianqian Ding, Alex F. Chen, Meng Xiang, Sifeng Chen

**Affiliations:** 1 Department of Physiology and Pathophysiology, School of Basic Medical Sciences, Fudan University, Shanghai, China

**Keywords:** aging, angiogenesis, exosomes, pluripotent stem cells

## Abstract

Heterochronic parabiosis has shown that aging individuals can be rejuvenated by a youthful circulatory system; however, the underlying mechanisms remain unclear. Here, we evaluated the effect of exosomes isolated from mouse induced pluripotent stem cells (iPSCs) on angiogenesis in naturally aged mice. To achieve this, the angiogenic capacity of aortic ring, the total antioxidant capacity (TAOC), p53 and p16 expression levels of major organs, the proliferation of adherent bone marrow cells, and the function and content of serum exosomes in aged mice administered iPSC-derived exosomes were examined. Additionally, the effect of iPSC-derived exosomes on injured human umbilical vein endothelial cells (HUVECs) was assessed. The angiogenic capacity of aortic rings and clonality of bone marrow cells from young mice were significantly higher than those from aged mice; moreover, the organs of aged mice had a higher expression of aging genes and lower total TAOC. However, *in vitro* and *in vivo* experiments showed that the administration of iPSC-derived exosomes significantly improved these parameters in aged mice. The synergistic effect of both *in vivo* and *in vitro* treatments of aortic rings with iPSC-derived exosomes improved the angiogenic capacity of aortic rings from aged mice to levels similar to that of young mice. Compared with untreated aged mice, serum exosomal protein content and their promoted effect on endothelial cell proliferation and angiogenesis were significantly higher in untreated young mice and aged mice treated with iPSC-derived exosomes. Overall, these results showed that iPSC-derived exosomes may rejuvenate the body by anti-aging the vascular system.

## INTRODUCTION

Aging is a naturally occurring lengthy biological process in humans, and is mostly associated with chronic, noncommunicable, disabling, and death-causing diseases [1, 2]. Strategies for decelerating, stopping, or reversing aging using therapeutic interventions have attracted considerable research attention. However, most studies on aging used genetic modified animal models, with only a few studies involving naturally aged animals. Moreover, genetic aging occurs only in approximately one in every 4 million newborns, whereas most individuals will encounter natural aging in later day of their life.

A decline in tissue regeneration potential and angiogenesis is a hallmark of aging, and natural aging decreases cell replacement and tissue regeneration [3, 4]. There is an inseparable relationship between organ and vascular aging, with vascular senescence being an important contributor to end-organ dysfunction [5]. Vascular aging can cause vascular dysfunction, which is the initiating factor of several cardiovascular diseases and the pathological basis of diseases, such as atherosclerosis and hypertension [6]. Recent findings suggest that young systemic milieu can rejuvenate aging organs [7]. Heterochronic parabiosis has shown that aging can affect stem cell population and angiogenesis. Transfusion with blood from young mice has been shown to increase the proliferation of stem cells in aged mice; moreover, serum from young animals stimulated endothelial cell proliferation by 88% compared with serum from old animals [8]. These findings suggest that intravascularly administered anti-aging substances may exert anti-aging effects.

Exosomes are bilipid membrane particles ranging from 40–160 nm in diameter, and are secreted by almost all cell types. Exosomes carry several proteins and RNAs that affect numerous pathways and are responsible for communication between cells [9]. Generally, the function of exosomes depends on their cell of origin, and exosomes from young blood may rejuvenate aging tissues via various processes. A significant advantage of exosomes is that they do not form teratomas [10, 11]. For example, intravenous administration of mesenchymal stem cell (MSC)-derived exosomes decreased myocardial and brain infarction areas [12]. Additionally, topical administration of stem cell-derived exosomes promoted wound healing and angiogenesis [13]. However, the effect of exosomes on naturally aged animals *in vivo* is unknown, especially the effect of induced pluripotent stem cell (iPSC)-derived exosomes on vascular proliferation and aging. Stem cells are the most active cell types in human, and have been reported to promote tissue repair [14]. Previous studies have shown that the administration of MSC- and iPSC-derived exosomes decreased myocardial infarction area and promoted cutaneous wound repair and angiogenesis [15, 16]. Given the size of exosomes, they cannot penetrate the intercellular gap between vascular endothelial cells to reach the parenchymal cells of solid organs. Therefore, we hypothesized that exosomes attached to and absorbed by endothelial cells exert anti-aging effects by increasing angiogenesis.

Angiogenesis is crucial for healthy blood microcirculation and is therefore a prerequisite for maintaining the revitalizing capacity of aging tissues. Persistently high P53 and P16 expression levels are associated with cell senescence [17]. Here, we evaluated the effect of exosomes isolated from mouse iPSCs on angiogenesis in naturally aged mice. To achieve this, the angiogenesis capacity of the aortic ring, the total antioxidant capacity (TOAC), the expression levels of p53 and p16, the clone number of adherent cells, and the function and components in aged mice administered iPSC-derived exosomes were examined. Then, exosomes from the serum of young mice and iPSC-derived exosomes were incubated with human umbilical vein endothelial cells (HUVEC) to determine whether cell proliferation and tube formation were promoted (Supplementary Figure 1).

## RESULTS

### iPSCs-derived exosomes promoted angiogenesis of aging aortic rings

**Figure 1:**
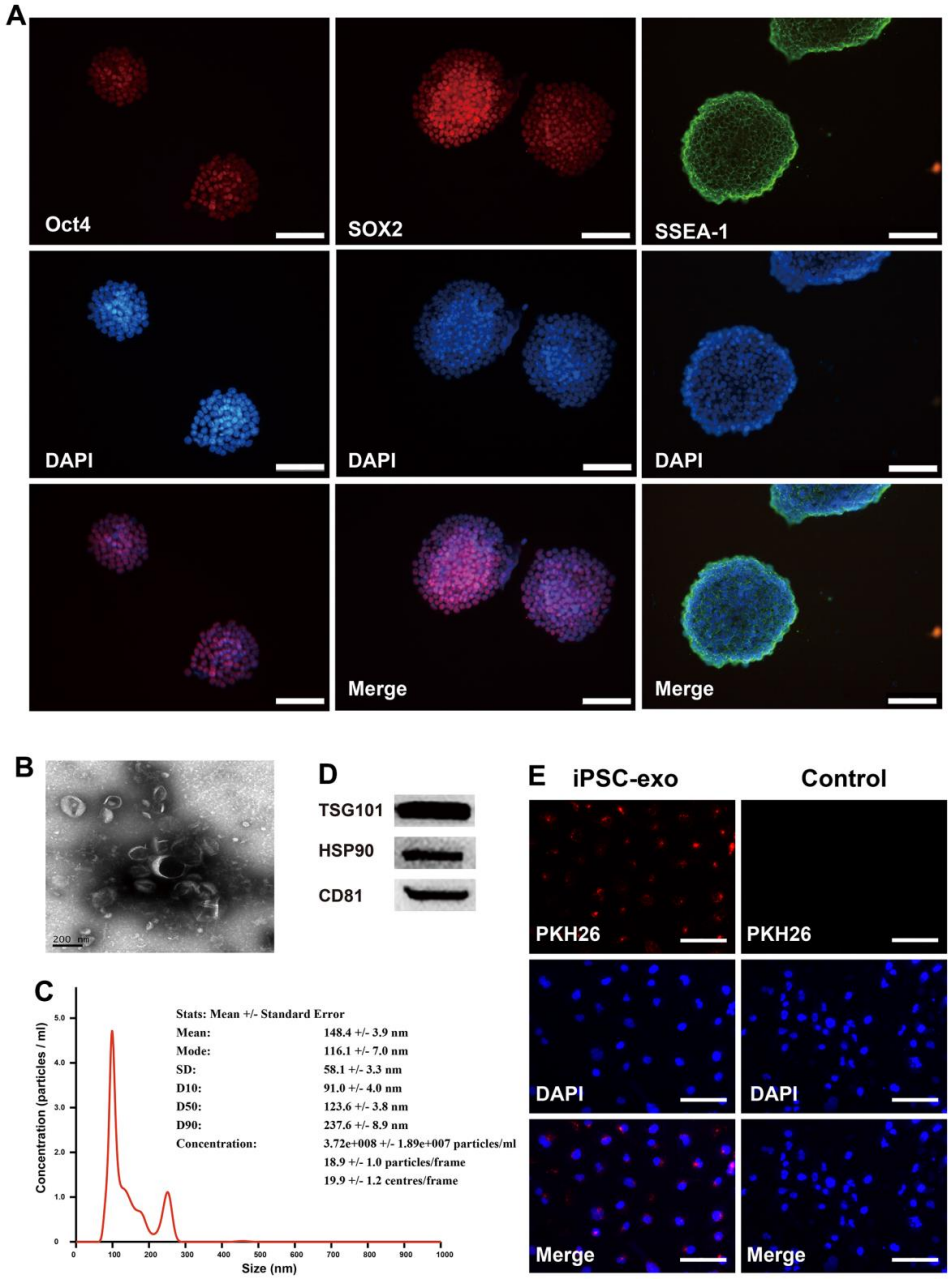
**Identification of mouse iPSCs and their exosomes.** (**A**) The cells were stained positive by immunofluorescence technique using specific antibodies for Oct4, SOX2 and SSEA-1. Oct4, octamer-binding transcription factor 4; SOX2, SRY-box transcription factor 2; SSEA-1, stage-specific embryonic antigen-1. (**B**) Transmission electron microscopy of iPSC-derived exosomes. (**C**) The concentrations and particle size of the exosomes were determined using nanoparticle tracking analysis. (**D**) Western blotting analysis was performed to detect the expression TSG101, HSP90, and CD81 in iPSC-derived exosomes. (**E**) Microscopic analysis of iPSC-derived exosomes taken up by HUVECs. Red, iPSC-derived exosomes labeled with PKH26; blue, the nuclei of HUVECs were counter-stained with DAPI. iPSCs, mouse induced pluripotent stem cells; exo, exosomes. TSG101, tumor susceptibility gene 101 protein; heat shock protein 90; CD31, cluster of differentiation molecule 31.

**Figure 2:**
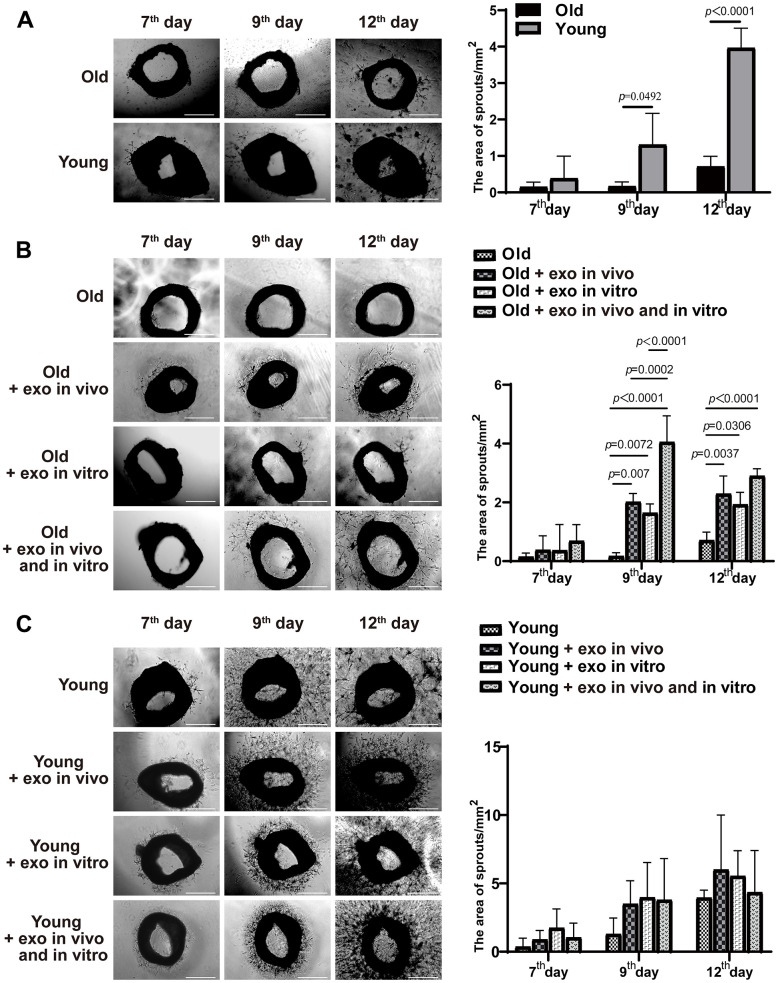
**iPSC-derived exosomes significantly increased the neovascularization of aortic rings in old mice.** (**A**) The accumulated neovascularized area of the aortic rings of untreated young mice were significantly higher than that of untreated old mice. (**B**) Effects of iPSC-derived exosomes on angiogenesis of aortic ring in old mice. Old: the aortic rings of untreated old mice; Old + exo *in vivo*: the aortic rings of old mice pre-treated with iPSC-derived exosomes by tail vein injection; Old + exo *in vitro*: the aortic rings of old mice were cultured with iPSC-exosomes *in vitro*; Old + exo *in vivo* and exo *in vitro*: the aortic rings of old mice pre-treated with iPSC-derived exosomes via tail vein injection were cultured with iPSC-derived exosomes *in vitro*. (**C**) Effects of iPSC-exosomes on angiogenesis of aortic ring in young mice. Young: the aortic rings of untreated young mice; Young + exo *in vivo*: the aortic rings of young mice pre-treated with iPSC-derived exosomes via tail vein injection; Young + exo *in vitro*: the aortic rings of untreated young mice were cultured with iPSC-exosomes *in vitro*; Young + exo *in vivo* and exo *in vitro*: the aortic rings of young mice pre-treated with iPSC-derived exosomes via tail vein injection were cultured with iPSC-exosomes *in vitro*. Scale bar, 500 μm. Quantification of the area of aortic ring is presented in the right panel. iPSCs, induced pluripotent stem cells; exo, exosomes.

Mouse iPSCs were generated by transducing mouse skin fibroblasts and confirmed by detecting the expression of the pluripotency markers Oct4, SOX2, and SSEA-1 (Figure 1A). Exosomes from iPSCs culture medium were isolated and characterized using transmission electron microscopy, nanoparticle tracking analysis (NTA), western blotting, and immunofluorescence assay (Figure 1B–1E). Aged and young mice were injected with iPSC-derived exosomes for three months via the tail vein, and neovascularized area of aortic rings were examined. New blood vessels were observed around the aortic rings of aged mice treated with iPSC-derived exosomes after 7 d of treatment and peaked after 12 d, followed by neovascularization and apoptosis. Compared with untreated young mice, there was a significant decrease in linear angiogenesis around the aortic rings in untreated old mice at 9 d and 12 d (Figure 2A). At 9 and 12 d of treatment, there was a significant increase in capillary-like sprout area around aortic rings of aged mice in the iPSC-exosome group (old + exo *in vivo*), in the vitro culture group (old + exo *in vitro*), and in the iPSC-exosomes group (old + exo *in vivo* + exo *in vitro*) compared with the untreated group, with linear angiogenesis appearing around the rings and gradually increasing to form a network structure. The angiogenesis area in the iPSC-exosomes group (old + exo *in vivo* + exo *in vitro*) was significantly larger than those of the iPSC-exosome pretreatment (old + exo *in vivo*) and *in vitro* culture groups (old + exo *in vitro*) at 9 d after treatment (Figure 2B). Additionally, neovascularization increased significantly at 7–12 d in the young group; however, there was no significant difference in the cumulated capillary-like sprouts area between the treatment groups at the same time points (Figure 2C).

### iPSCs-derived exosomes increased the clonality and proliferation of bone marrow cells

**Figure 3:**
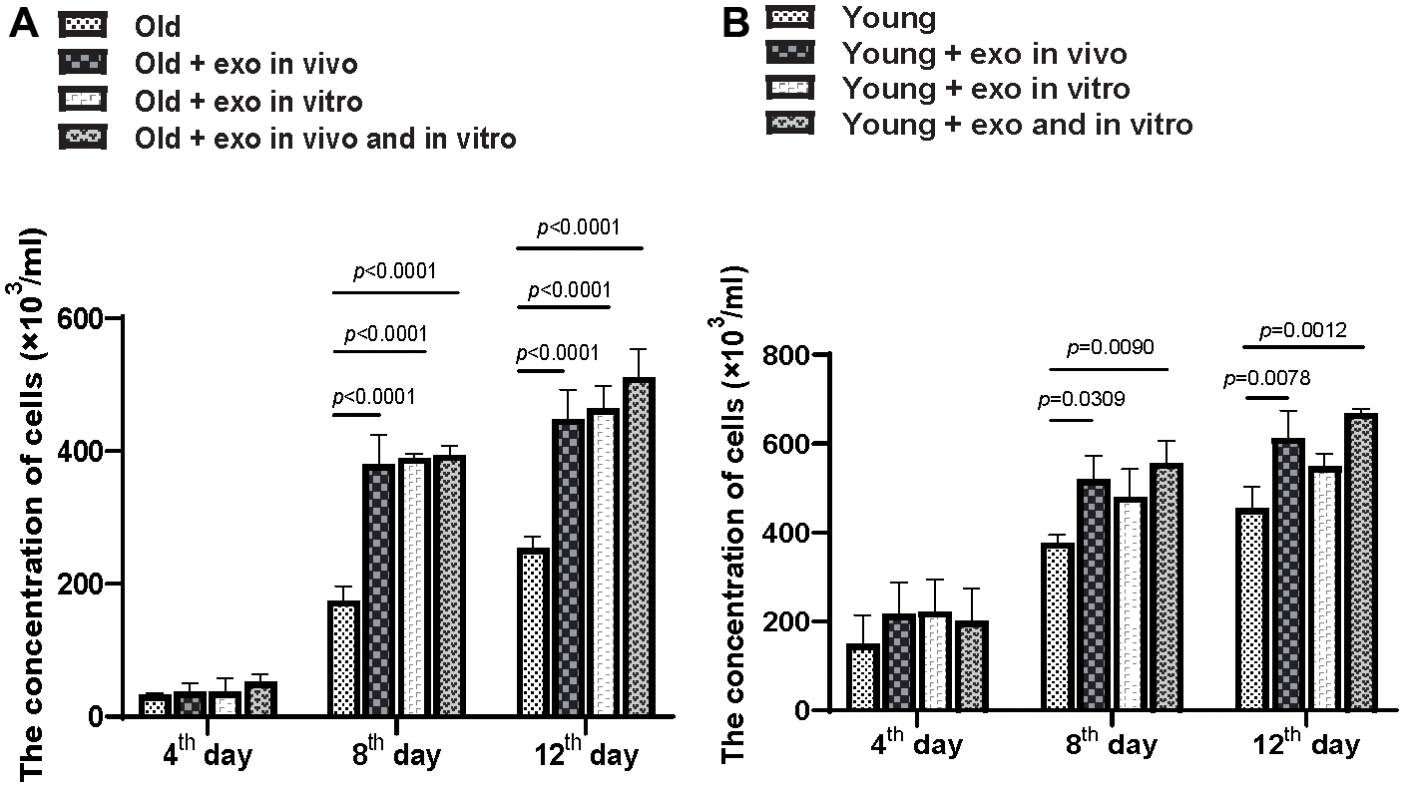
**iPSC-derived exosomes significantly improved BMC proliferation.** (**A**) Quantification of the concentration of BMCs from old mice in different treatment groups. Cloned BMCs were digested and calculated at 4, 8, and 12 d after treatment. Old: BMCs of untreated old mice; Old + exo *in vivo*: BMCs of old mice pre-treated with iPSC-derived exosomes via tail vein injection; Old + exo *in vitro*: BMCs of old mice were cultured with iPSC-exosomes *in vitro*; Old + exo *in vivo* and exo *in vitro*: BMCs of old mice pre-treated with iPSC-derived exosomes via tail vein injection were cultured with iPSC-exosomes *in vitro*. (**B**) Quantification of the concentration of BMCs from young mice with different treatment groups. Cloned BMCs were digested and calculated 4, 8, and 12 d after treatment. Young: BMCs of untreated young mice; Young + exo *in vivo*: BMCs of young mice pre-treated with iPSC-derived exosomes via tail vein injection; Young + exo *in vitro*: BMCs of untreated young mice were cultured with iPSC-exosomes *in vitro*; Young + exo *in vivo* and exo *in vitro*: BMCs of young mice pre-treated with iPSC-derived exosomes via tail vein injection were cultured with iPSC-exosomes *in vitro*. iPSCs, induced pluripotent stem cells; exo, exosomes; BMCs, bone marrow stromal cell.

Adherent bone marrow cells were isolated from all mice and cultured, and the number of bone marrow cells in old and young mice was counted. Exosomes treatment increased the clonality of bone marrow cells from aged mice after 12 d regardless of whether the exosomes were cultured *in vitro* or pre-injected *in vivo*. Additionally, *in vivo* or *in vitro* administration of iPSC-exosomes significantly increased the proliferation of bone marrow cells from aged mice at 8 and 12 d of treatment but not at 4 d (Figure 3A). Compared with the old untreated group, *in vitro*, *in vivo*, and *in vivo* + *in vitro* treatment significantly increased cell concentrations at 8 and 12 d after treatment. In the case of bone marrow cells from young mice (Figure 3B), *in vivo* and *in vivo* + *in vitro* treatments with exosomes increased clonality at 8 d and 12 d after treatment (Figure 3B). However, *in vitro* exosome treatment did not significantly affect clonality at 8 or 12 d after treatment.

### Intravenous administration of exosomes inhibited aging in old mice

**Figure 4:**
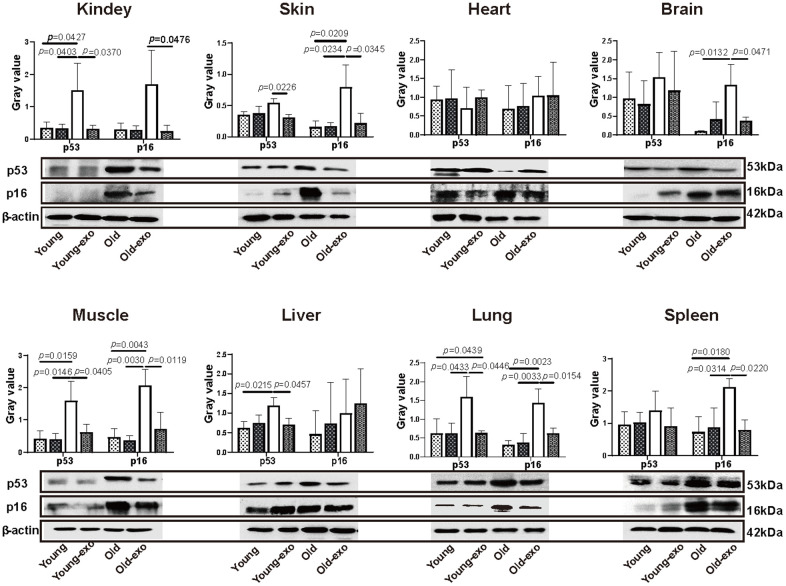
**Treatment with iPSC-derived exosomes significantly decreased p53 and P16 expression levels in organs of old mice.** Western blot images of p53 and p16 expression in heart, liver, spleen, lung, kidney, brain, skin, and muscle of mice in different treatment groups, and the quantification of the western blot results. β-actin was used as an internal control. Young: untreated young mice; young + exo: young mice pre-treated with iPSC-derived exosomes via tail vein injection; Old: untreated old mice; Old + exo: old mice pre-treated with iPSC-derived exosomes via tail vein injection. iPSCs, induced pluripotent stem cells; exo, exosomes.

Angiogenesis is necessary for the repair of parenchymal organs and to slow tissue aging. Persistently high p53 and p16 expression has been shown to promote cell senescence. Compared with the young mice, p53 expression was significantly higher in the kidney, muscle, liver, and lung tissues of aged mice (Figure 4). Intravenous injection of iPSC-exosomes for three months significantly decreased p53 expression levels in the kidney, skin, muscle, liver, and lung tissues of aged mice compared with that of untreated aged mice. Similarly, p16 protein expression was significantly higher in the skin, brain, muscle, lung, and spleen of aged mice than in the organs of young mice. Furthermore, intravenous injection of iPSC-exosomes for three months significantly decreased p16 expression in the kidney, skin, brain, muscle, lung, and spleen tissues of aged mice compared with that of untreated aged mice. However, there were no significant differences in p53 and p16 expression levels between untreated young mice and young mice administered iPSC-exosomes (Figure 4).

### Intravenous administration of exosomes increased total antioxidant activity in tissues of aged mice

**Figure 5:**
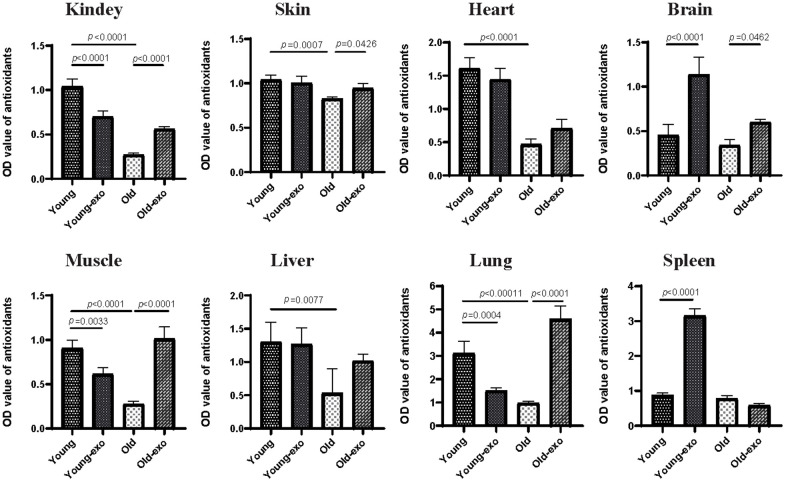
**Treatment with iPSC-derived exosomes increased total antioxidant capacity (TAOC) in organs of old mice.** The TAOC of heart, liver, spleen, lung, kidney, brain, skin, and muscle were measured using ABTS kit. Young: untreated young mice; young + exo: young mice pre-treated with iPSC-derived exosomes via tail vein injection; Old: untreated old mice; Old + exo: old mice pre-treated with iPSC-derived exosomes via tail vein injection. TAOC, the total antioxidant capacity; iPSCs, induced pluripotent stem cells; exo, exosomes.

Healthy tissues and organs contain several antioxidative molecules and enzymes that can eliminate reactive oxygen species (ROS) to prevent oxidative stress. Therefore, the effect of exosome treatment on the TAOC of the kidney, skin, heart, brain, muscle, liver, spleen, and lungs of mice was examined using ABTS kit. The TAOC of the kidney, skin, heart, muscle, liver, and lung tissues of aged mice was significantly lower than that of young mice. Compared with untreated aged mice, treatment with iPSC-exosome increased the TAOC of the kidney, skin, brain, muscle, and lung tissues of aged mice by varying degrees (Figure 5).

However, the effect of iPSC-exosome on young mice varied among the organs; specifically, the TAOC of the brain and spleen tissues of exosome-treated young mice was significantly higher than those of untreated young mice. In contrast, the TAOC of the kidney, muscle, and lung of exosome-treated young mice was significantly lower than those of untreated young mice; however, there were no significant differences in the TAOC in liver, skin and heart between the groups. Regardless of iPSC-exosome treatment, TAOC was higher in young mice than in untreated old mice (Figure 5).

### iPSC-derived exosomes or serum exosomes from mice treated with iPSC-derived exosomes decreased HUVEC injury

**Figure 6:**
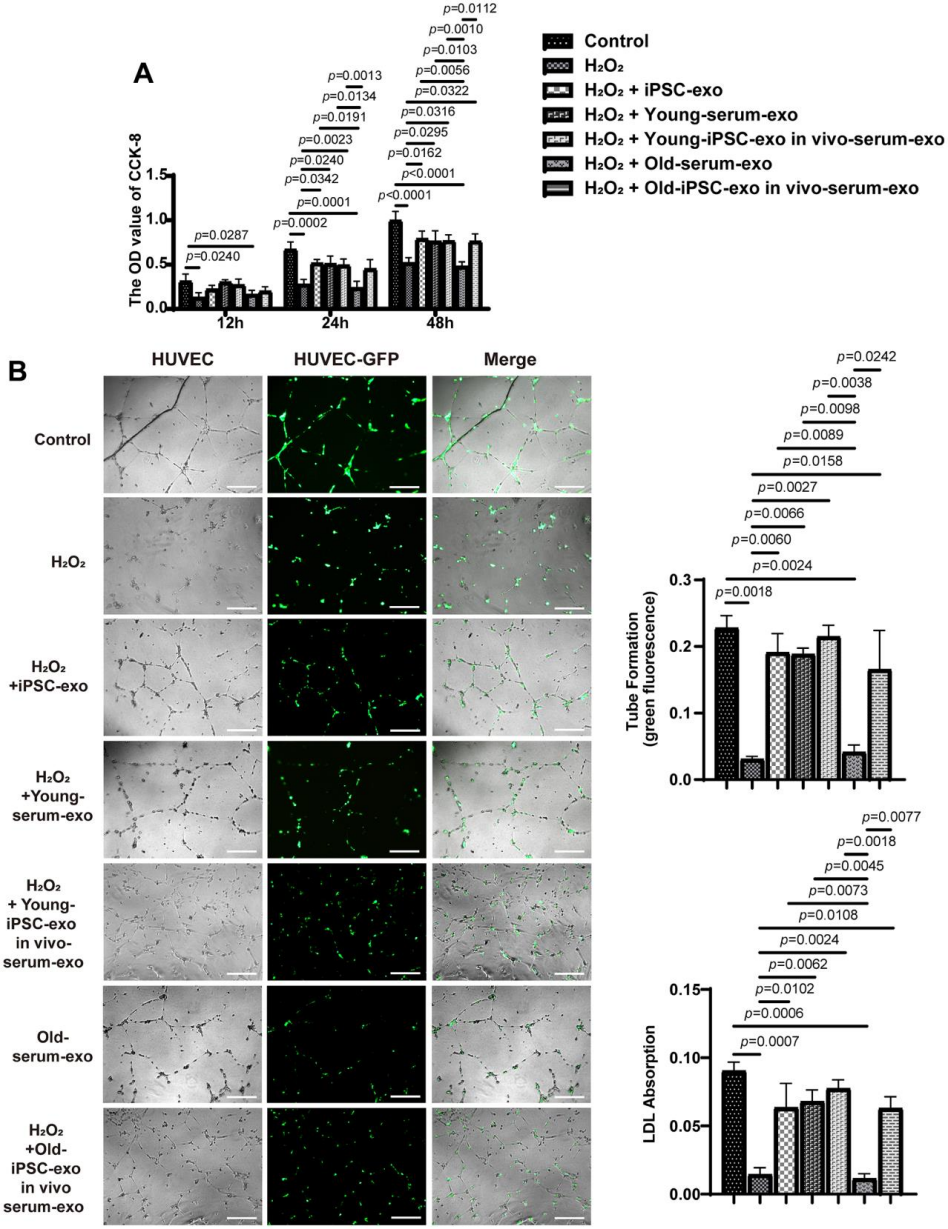
**Treatment with iPSC-derived exosomes and serum exosomes of young mice restored endothelial function.** (**A**) iPSC-derived exosomes and serum exosomes of young mice promoted the proliferation of HUVEC. The proliferation of HUVEC was detected using CCK8 kit after H_2_O_2_ injury. (**B**) iPSC-derived exosomes and serum exosomes of young mice restored the ability of endothelial cells to form tube after H_2_O_2_ injury. Green fluorescence was observed to measure the tube formation by endothelial cells. HUVEC, human umbilical vein endothelial cell; LDL, low density lipoprotein; AC-LDL, acetylated low density lipoprotein.

The effect of iPSC-derived exosomes and serum exosomes from aged mice treated with iPSC-derived exosomes (old-serum-exo) on HUVEC with H_2_O_2_ to induce endothelial cell injury was examined at 12, 24, and 48 h after co-culture (Figure 6A). Compared with the untreated H_2_O_2_ injured group, treatment with serum exosomes from untreated or iPSC-derived exosomes treated young mice, as well as iPSC-exosomes, promoted the proliferation of injured HUVEC after 24 and 48 h of co-culture. Similarly, serum exosomes from old mice treated with the iPSC-derived exosomes promoted the proliferative ability of injured cells at 48 h. Additionally, treatment with iPSC-derived exosomes or serum exosomes of young mice also increased the ability of endothelial cells to form capillary-like structure and to uptake acetylated low density lipoprotein (Figure 6B). Overall, these results indicated that iPSC-derived exosomes or serum exosomes from mice treated with iPSC-derived exosomes can restore endothelial function.

### Protein profiles of exosomes

Exosomes have been confirmed to play a variety of roles mainly due to their source and the selective encapsulation of a variety of functional proteins. Therefore, we examined the protein profiles of serum exosomes of untreated old mice (old-serum-exo), old mice treated with iPSC-exosomes (old+iPSC-exo-serum-exo), untreated young mice (young-serum-exo), and young mice injected with iPSC-exosomes (young+iPSC-exo-serum-exo), using liquid chromatography with tandem mass spectrometry (LC-MS/MS). Additionally, bioinformatics was performed to identify differentially expressed proteins, followed by functional annotation and pathway enrichment analysis of the proteins using gene ontology (GO) and Kyoto Encyclopedia of Genes and Genomes databases (KEGG).

**Figure 7:**
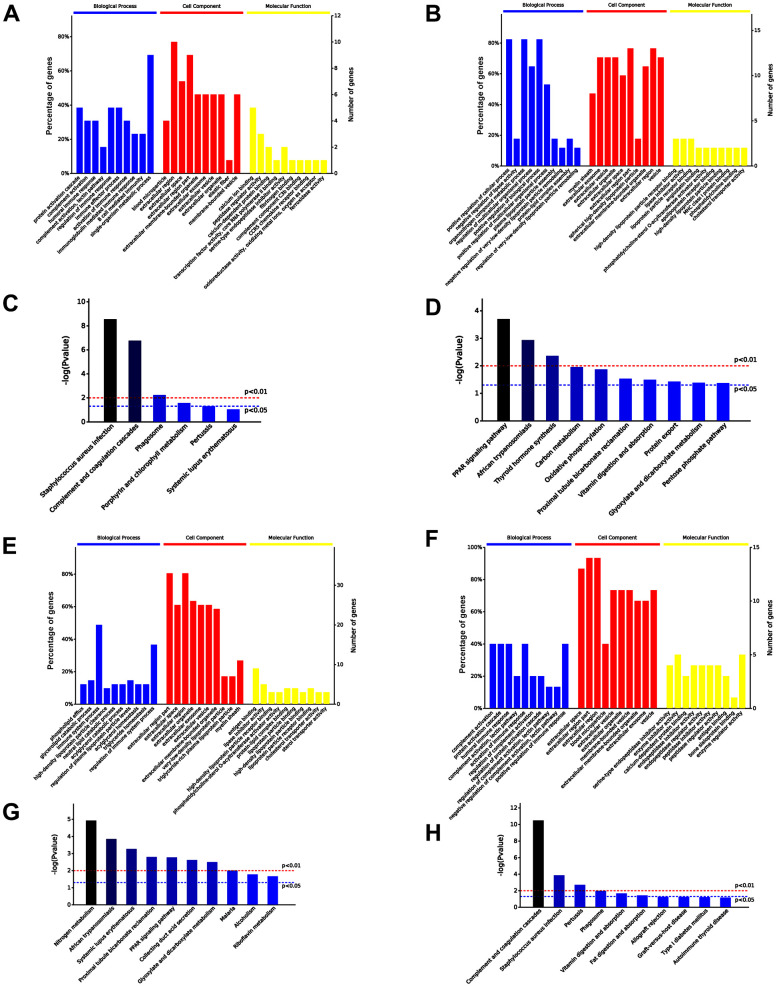
**Comparison of serum exosomal proteins between old and young mice groups.** (**A**, **B**) Gene ontology (GO) functional annotation of up- and down-regulated exosomal proteins in old serum group, respectively. (**C**, **D**) Kyoto Encyclopedia of Genes and Genomes (KEGG) pathway enrichment analysis of upregulated and downregulated exosomal proteins in old serum group. (**E**) GO functional annotation of upregulated exosomal proteins in old mice pre-injected with iPSC-exosomes. (**F**) GO functional annotation of the downregulated exosomal proteins in old mice pre-injected with iPSC-exosomes. (**G**) KEGG pathway enrichment analysis of upregulated exosomal proteins of old mice pre-injected with iPSC-exosomes. (**H**) KEGG pathway enrichment analysis of downregulated exosomal proteins of old mice pre-injected with iPSC-exosomes. BP, biological process; MF, molecular function; CC, cellular component.

A total 30 proteins were differentially expressed in serum exosomes of untreated aged and young mice, among which 13 proteins were upregulated and 17 downregulated in serum exosomes of untreated aged mice. The top 10 significantly enriched GO terms by the 30 proteins are presented in Figure 7A, 7B. Specifically, the upregulated proteins were enriched in immune response and metabolic process related to higher immune/inflammatory response, while the downregulated proteins were involved in positive regulation of biological process and metabolic process related to increased cell vitality and decreased blood lipid levels in the “Biological Processes” (BP) category. Additionally, the upregulated proteins were enriched in calcium ion binding, while the downregulated proteins were enriched in lipid binding, including high-density lipoprotein particle receptor binding, in the “Molecular Function” (MF) category. Moreover, KEGG analysis revealed that the upregulated proteins were involved in complement and coagulation cascades, while the downregulated proteins were involved in PPAR signaling pathway (Figure 7C, 7D).

Furthermore, a total of 56 differentially expressed proteins were identified in the serum exosomes of young mice treated with iPSC-exosomes compared with the levels in the exosomes of old mice treated with iPSC-exosomes group, among which 41 were upregulated and 15 were downregulated in the exosomes of old mice treated with iPSC-exosomes. The top 10 significantly enriched GO terms are presented in Figure 7E, 7F. Specifically, the upregulated proteins were enriched in immune system response, while the downregulated proteins were enriched in immune system response, including complement activation and humoral immune response, in the BP category. Additionally, the upregulated proteins were enriched in antigen binding, while the downregulated proteins were enriched in enzyme regulator activity in the BP category. KEGG enrichment analysis showed that the upregulated and downregulated proteins were mainly enriched in nitrogen metabolism and complement and coagulation cascades, respectively (Figure 7G, 7H).

**Figure 8:**
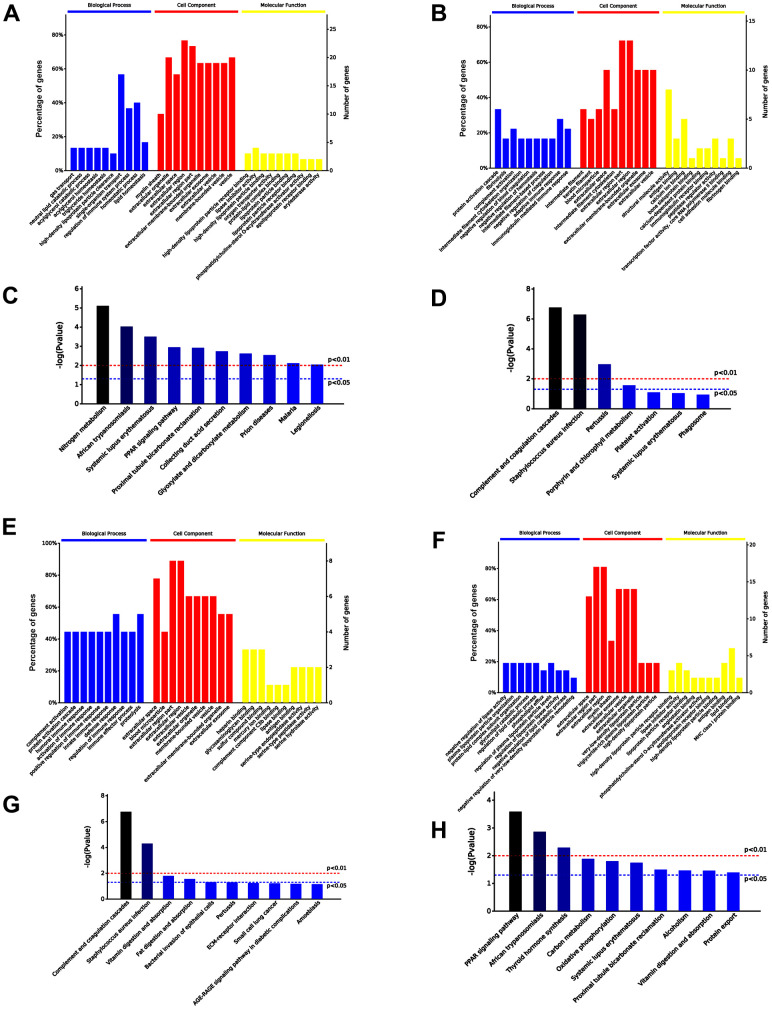
**Comparison of serum exosomal proteins between untreated old mice and old mice pre-injected with iPSC-exosomes, and between untreated young mice and young mice pre-injected with iPSC-exosomes groups.** (**A**, **B**) GO functional annotation of the up- and the down-regulated exosomal proteins in old mice pre-injected with iPSC-exosomes, respectively. (**C**, **D**) KEGG pathway enrichment analysis of up- and down-regulated exosomal proteins in old mice pre-injected with iPSC-exosomes, respectively. (**E**, **F**) GO functional annotation of the up- and the down-regulated exosomal proteins in young mice pre-injected with iPSC-exosomes group, respectively. (**G**, **H**) KEGG pathway enrichment analysis of up- and down-regulated exosomal proteins of young mice pre-injected with iPSC-exosomes group. BP, biological process; MF, molecular function; CC, cellular component.

Among 48 differentially expressed proteins, 30 and 18 were significantly upregulated and downregulated, respectively, in the serum exosomes of old mice treated with iPSC-exosomes compared with the expression levels in the serum exosomes of untreated old mice. GO functional annotation showed that the 30 upregulated proteins were enriched in biological processes that are more active in vigorous cells, while the 18 downregulated proteins were enriched in metabolic processes and immune response in the BP category. Additionally, the upregulated proteins were enriched in binding (such as high-density lipoprotein particle receptor binding) and enzyme regulator activity, while the downregulated proteins were enriched in protein binding in the MF category. KEGG pathway analysis showed that the upregulated proteins were involved in nitrogen metabolism, while the downregulated proteins were enriched in complement and coagulation cascades (Figure 8A–8D). Additionally, nine proteins were upregulated and 21 were downregulated in the serum exosomes of aged mice treated with iPSC-exosomes compared with the expression levels in the serum exosomes of untreated young mice. GO functional annotation showed that the upregulated proteins were enriched in immune system response, while the downregulated 21 proteins were enriched in negative regulation of lipase activity and lipid catabolic process in the BP category. Additionally, the upregulated proteins were enriched in binding of many molecules, while the downregulated proteins were enriched lipid binding in the MF category. KEGG pathway analysis showed that the upregulated proteins were enriched in complement and coagulation cascades, while the downregulated proteins were enriched in PPAR signaling pathway (Figure 8E–8H). Moreover, all proteins were enriched in exosome-related components in the “Cellular Components” (CC) category, which further proved that the extracted proteins were exosome components.

**Figure 9:**
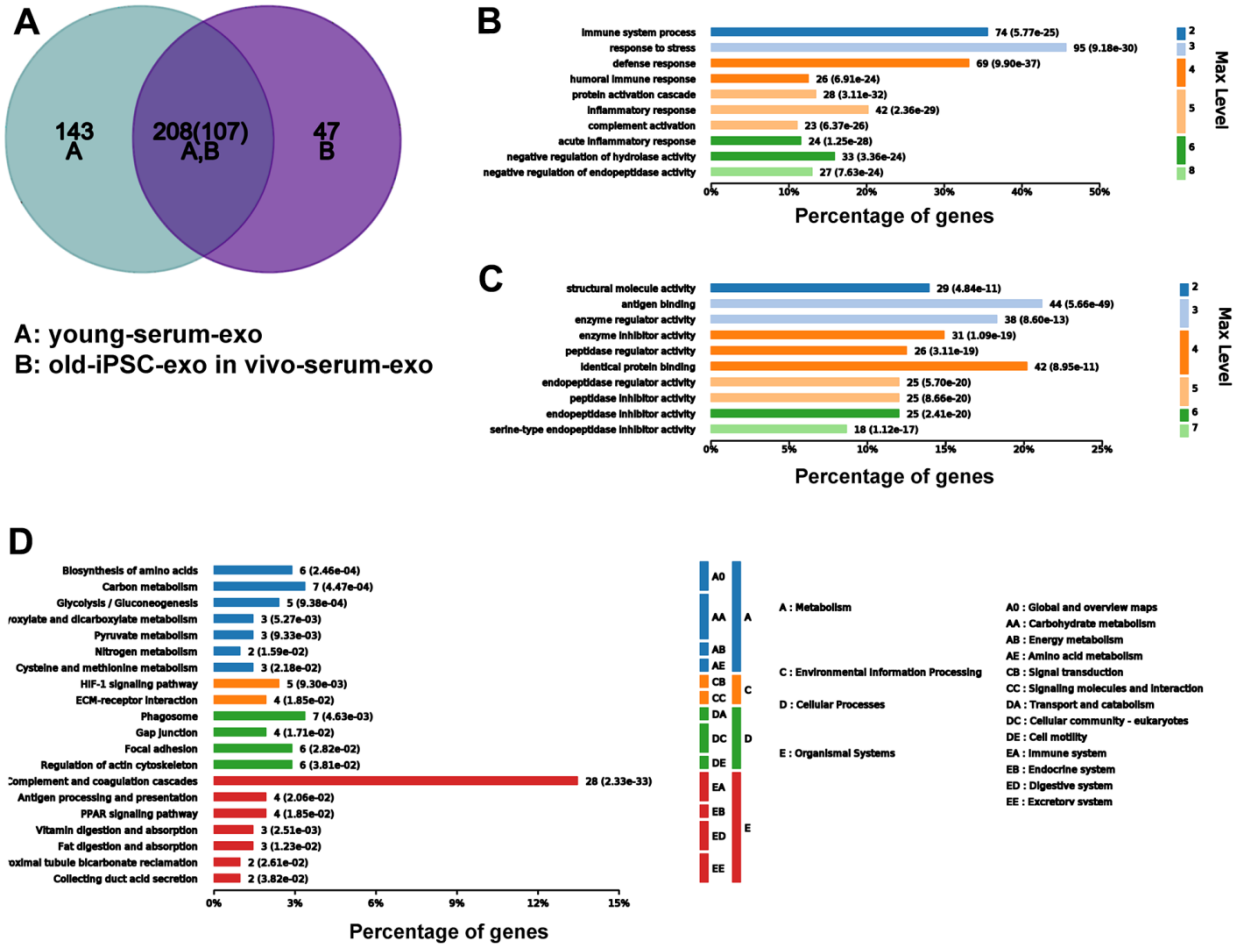
**Serum exosomal protein profile of old mice pre-injected with iPSC-exosomes was similar to that of young mice in the untreated group.** (**A**–**D**) Venn diagram, and GO and KEGG enrichment analyses of the common exosomal proteins in serum of old mice pre-injected with iPSC-exosomes and untreated mice.

To further demonstrate that serum exosomes of aged mice injected with iPSC-exosomes played a similar role to the serum exosomes of untreated young mice, Venn diagram showed that 208 differentially expressed proteins were common to both groups (Figure 9A). GO functional annotation showed that the proteins were enriched biological processes related to stronger responses to stress and immune/inflammatory injury in the BP category, and in antigen binding and inhibitor activity of key metabolic enzymes in the MF category (Figure 9B, 9C). KEGG signaling pathway showed that proteins were enriched in several pathways related to cellular metabolisms and complement and coagulation cascade (Figure 9D).

### No teratoma in experiment mice

After sample harvesting, the whole body of the animals were examined morphologically to determine if there was any teratoma in the animals. Teratoma was not observed in the body of the experimental animals.

## DISCUSSION

Aging is associated with interconnected processes, including genomic instability, telomere attrition, epigenetic alterations, loss of proteostasis, impaired macro-autophagy, deregulated nutrient sensing, mitochondrial dysfunction, cellular senescence, stem cell exhaustion, altered intercellular communication, chronic inflammation, and dysbiosis [18]. Vascular aging is a critical prepositional factor for vascular diseases that can lead to organ aging. Aging vessels are vulnerable to harmful circulatory factors and difficult to regenerate. The vascular circulation connects all organs and transfers aging-inducing factors to these organs. The inability of the vascular endothelium to provide effective and timely defense against harmful stimuli can lead to vascular lesions [19]. In contrast, the timely repair of damaged blood vessels is necessary to restore blood supply to organs and tissues, which may prevent organ and tissue injury or even reverse organ injury [20].

In the present study, the aortic ring model was used to determine the effect of iPSC-derived exosomes on vascular proliferation. Treatment with iPSC-derived exosomes significantly increased angiogenesis of aortic rings from aged mice regardless of whether the exosomes were cultured *in vitro* or pre-injected *in vivo*. Additionally, iPSC-derived exosome-induced angiogenesis did not exceed normal level because the exosomes did not promote angiogenesis of aortic rings from young mice. Moreover, the exosome-induced angiogenesis of aortic rings from old mice was not as high as those of untreated aortic rings of young mice.

Clonality and proliferation of bone marrow cells are associated with ageing and regeneration. Stem cells released from bone marrow play an important role in regeneration of vasculature. Interaction between endothelium and stem cells facilitates selective extravasation and homing of stem cells. Additionally, the vasculature can influence stem cell proliferation and differentiation by providing molecules secreted by endothelial cells. Moreover, the number of bone marrow stem cells (BMCs) is higher in young mice than in old mice [21]. In the present study, exosome treatment increased the number of BMC clones regardless of whether the exosomes were cultured *in vitro* or pre-injected *in vivo*. Bone vasculature is composed of a fenestrated capillary, and exosomes may pass through the pores of the fenestrated capillary entrance into the bone marrow to increase the clonality of BMCs. Treatment with BMCs may release more youthful factors to delay the aging process of related organs.

Oxidative stress has been identified as the leading cause of tissue aging [22]. Stem cell-derived exosomes have been shown to reduce oxidative stress in ischemic tissues by increasing ATP and NADH levels [23, 24]. Similarly, iPSC-derived exosomes enhanced TAOC of the organs of aged mice, including the skin, heart, brain, muscle, lung, and kidney, indicating that stem cell-derived exosomes may delay aging to some extent by increasing TAOC. ROS-induced cell aging has been shown to upregulate the expression of senescence markers p53 and p16 [25]. In the present study, treatment with iPSC-derived exosomes significantly reduced p53 and p16 expression in old mouse, indicating that exosomes can reduce oxidative stress, ameliorate ROS-induced cell damage, and delay tissue aging to some extent by increasing the TAOC of various tissues. However, the effect of iPSC-derived exosomes on TAOC in young mice varied between organs, which could be because the additional antioxidant activity provided by exosomes is not necessary in young mice.

Previous studies have shown that youthful circulating factors can restore the self-renewal and differentiation potential of aged stem cells. Some "young factors" present in the serum of young mice have been reported to reverse the aging of old mice, but the exact mechanism is not clear. Yoshida et al. reported that extracellular vesicles containing nicotinamide phosphoribosyltransferase (eNAMPT) isolated from young mice significantly improved body function and extended the lifespan of older mice [26]. In the present study, iPSC-derived exosomes increased vascular regeneration and clone formation in mesenchymal stem cells of old mice. In young mice, stem cell-derived exosomes are taken up by endothelial cells and function as serum exosomes. To further verify that iPSC-derived exosomes can promote vascular endothelium regeneration, H_2_O_2_-injured HUVEC were treated with iPSC-derived exosomes, and the proliferation and formation of capillary-like structures in HUVEC were examined. Treatment with iPSC-derived exosomes significantly promoted the proliferation and length of tube formation in HUVEC. The results confirmed that iPSC-derived exosome-induced improvement in vascular regeneration and clonality of stem cells is necessary in delaying aging.

Huang, et al. found differential effects of extracellular vesicles from aging and young mesenchymal stem cells in acute lung injury [27]. In the present study, iPSC-derived exosomes increased the clonality and proliferation of bone marrow cells. Thus, it is possible that serum exosomes may change due to iPSC-exosome treatment. Accordingly, we speculated that iPSC-derived exosomes may be absorbed and induce the formation of new exosomes in the blood circulation, and that the function of the new exosomes would be similar to that of the active proteins in the serum exosomes of young mice. Proteomic analysis confirmed that the protein profile of serum exosomes from old mice treated with iPSCs-exosomes was similar to that serum exosomes from untreated young mice. Additionally, Venn analysis indicated that 208 proteins were co-expressed in serum exosomes of old mice pre-injected with iPSC-exosomes and untreated young mice. GO and KEGG analysis showed that the proteins were mainly involved in vigorous cell metabolism and improved defense capability. Overall, these results indicated the serum exosomal component was rejuvenated by iPSC-derived exosomes, which may be helpful for rejuvenating the whole animal, and may serve as an indicator of a rejuvenated animal.

Teratoma formation is a concern of cell therapy using pluripotent stem cells and their derivatives. All published articles including several articles of ours have shown that iPSC-derived exosomes did not induce teratoma *in vivo*. Similarly, treatment with iPSC-derived exosomes did not induce teratoma in the mice in the present study (data not shown). Moreover, pluripotent factors were not identified in iPSC-derived exosomes as shown in the Supplementary Files of a previous study [28].

Conclusively, treatment with exosomes derived from iPSCs isolated from young mice improved serum exosomal component, the total antioxidant capacity of several organs, reduced the expression of aging markers, increased angiogenesis of aortic rings, and promoted the clonality of MSCs in old mice. Overall, the exosomes exerted antiaging effects by promoting angiogenesis in mice.

## MATERIALS AND METHODS

### iPSCs culture and characterization

iPSCs were induced from the skin fibroblast of C57 mouse as described previously [29]. Briefly, iPSCs were recovered a medium containing Dulbecco’s modified eagle medium (DMEM; Thermo Fisher Scientific, Waltham, MA, USA), 10% fetal bovine serum (FBS; Thermo Fisher SCientific, Waltham, MA, USA), 1% non-essential amino acid (NEAA; Thermo Fisher Scientific, Waltham, MA, USA), 1% penicillin and streptomycin (PS; Thermo Fisher Scientific, Waltham, MA, USA), 0.01% CHIR98014 (Selleck Chemicals, Houston, TX, USA), 0.01% mirdametinib (PD0325901; Selleck Chemicals, Houston, TX, USA), and 1 × 10^7^ U/mL of recombinant murine leukemia inhibitory factor (mLIF; Peprotech, Rocky Hill, NJ). The culture medium for iPSCs was changed to a medium containing 90% DMEM, 10% knockout serum replacements (KSR; Thermo Fisher Scientific, Waltham, MA, USA), 1% NEAA, 1% PS, 0.01% CHIR98014, 0.01% PD0325901, and 1 × 10^7^ U/mL of mLIF 1 d after cell recovery.

The medium containing KSR was collected daily for exosome purification. iPSCs were fixed in 4% paraformaldehyde at 37° C for 15 min and then incubated with 5% donkey serum and 0.3% Triton X-100 for 60 min. The cells were incubated with the pluripotency markers octamer-binding transcription factor 4 (Oct4), sex-determining region Y-box 2 (SOX2), or Stage-Specific Embryonic Antigen-1 (SSEA-1) (rabbit anti-mouse, 1:1000; Abcam, Cambridge, UK) at 4° C overnight. Thereafter, the cells were washed and incubated with secondary antibodies at 22° C for 60 min, followed by staining with DPAI.

### Exosome extraction, identification, and storage

Cell culture media were centrifuged at 200 *g* for 10 min and then at 2000 *g* for 20 min at 4° C to remove debris and dead cells. Subsequently, the cell-free culture supernatant (4 mL) was incubated with 1 mL of ExoQuick-TC solution (System Biosciences, Palo Alto, CA, USA) overnight at 4° C, and exosomes were isolated by centrifugation at 1500 *g* for 30 min. The precipitates were resuspended in 1 mL of PBS, further purified by ultracentrifugation at 100,000 *g* for 70 min, and finally resuspended in 100 μL of PBS. Exosomes were detected using high-resolution transmission electron microscopy and nanosight tracking analysis (NTA; NanoSight NS300, UK). The expression of TSG101 and CD63 (specific protein markers of exosomes) were determined using western blotting analysis and the respective primary antibodies TSG101/HSP90/CD63 (rabbit anti mouse, 1:1000; Abcam, Cambridge, UK) and secondary antibody IgG (donkey anti rabbit, 1:10000; Jackson Laboratory, Bar Harbor, ME, USA). The isolated exosomes were stored at -80° C until use. For the uptake assay, exosomes labeled with PKH26 (Sigma-Aldrich, Merck KGaA, Germany) were incubated with HUVECs for 24 h and nuclei were counterstained with DAPI (blue), and the cells were then photographed.

### Animal arrangement

The animal protocol was approved by the University of Fudan Animal Care Committee in conformity with the Guide for the Care and Use of Laboratory Animals (National Research Council of United States). The mice were maintained on a standard rodent chow and water *ad libitum*. A total of 12 mice comprising of six aged mice (42 months old) and six young mice (6–8 weeks) were used for this study. Three aged and three young mice were injected with PBS via the tail vein once every 3 d for three months as the control groups, while the remaining three aged and three young mice were injected with exosomes derived from iPSCs (doses 200 μg) for 3 months as the experimental group.

The *in vitro* experiments using aortic rings and nucleated bone marrow from aged and young mice were separately subdivided into four groups. Old mouse group: untreated group (old); *in vivo* pretreatment with iPSC-exosomes (old + exo *in vivo*); *in vitro* culture with iPSC-exosomes (old + exo *in vitro*); *in vivo* pretreatment and *in vitro* culture with iPSC-exosomes (old + exo *in vivo* and exo *in vitro*). Young mice: untreated group (young); *in vivo* pretreatment with iPSC-exosomes group (young + exo *in vivo*); *in vitro* culture with iPSC-exosomes group (young + exo *in vitro*); and *in vivo* pretreatment and *in vitro* culture with iPSC-exosomes group (young + exo *in vivo* and exo *in vitro*).

### Aortic ring extraction

Aortic ring extraction was performed according to previously described method [30]. Briefly, the ventral skin was opened with single cut and blunt dissected using dissection scissors. The thoracic cavity was cut open using fresh sterile scissors, and the heart and lungs were removed to expose the aorta along the spine. The anterior end of the aorta was gently grasped, and the aorta was separated from the spinal column by blunt dissection, ending before the artery branching into the iliacs in the abdomen. The dissected aorta was transferred into sterile PBS. The tunica adventitia of the artery was gently removed as cleanly as possible in sterilized PBS using micro-forceps sterilized in 75% alcohol for 1 h. The aorta pectoralis was placed in another dish with sterilized PBS and then cut into approximately 10 aortic rings using micro-scissors.

### Teratoma examination

After sample harvesting, the whole body of the animals were examined morphologically to determine if there was any teratoma in the animals.

### Aortic ring culture

Thawed Matrigel (No.356237; BD Biosciences, Bedford, MA, USA) (4 mg/mL) was diluted in DMEM (6 mg/mL), and 50 μL of diluted Matrigel was transferred into one well of 96-well plate. Subsequently, the 96-well plates were incubated in a cell incubator for 15 min. The ring was carefully transferred into Matrigel using micro-forceps, and 50 μL of additional diluted Matrigel was added into the well, followed by incubation for 15 min. Finally, 100 μL of ECM was added into the well to culture the aortic ring. Rings from each mouse were divided into two categories: ECM with exosomes (derived from iPSCs; total amount: 10 μg per well) and ECM without exosomes. The medium was changed after 5 d and then every 2 d, and the rings were continuously observed and photographed. All images were processed using ImageJ software (National Institutes of Health, Bethesda, MD, USA) to calculate the area of sprout vessels and the number of clones.

### Bone marrow cell extraction and culture

Bone marrow cells (BMCs) were isolated from the bone marrow of sacrificed mice and cultured in a medium containing 80% α-MEM and 20% FBS. The cells were treated with 10 μg of exosomes and the number of adherent cell clones was calculated at 4, 8, and 12 d after treatment. Cloned BMCs were digested with trypsin on 4, 8, and 12 d after treatment and the number of cells in each well was then counted using a cell counter.

### Tissues extraction and Western blotting

Twelve mice were sacrificed after three months, and skin, muscle, kidney, liver, spleen, lung, heart, and brain tissues were sampled using 1.5 mL tubes and stored in a refrigerator at -80° C. Proteins were extracted from the tissues using radioimmunoprecipitation (RIPA) buffer (Thermo Fisher Scientific, Waltham, MA, USA). Subsequently, the proteins were denatured, resolved on a 12% polyacrylamide gel, and transferred onto a polyvinylidene fluoride membrane (PVDF; Millipore, Bedford, MA, USA). The membranes were incubated overnight with primary antibodies against p53, p16, and β-actin (rabbit anti mouse, 1:1000; Abcam, Cambridge, UK) at 4° C. Thereafter, the PVDF membranes were washed with PBST (Thermo Fisher Scientific, Waltham, MA) three times for 15 min each, and incubated with donkey anti-rabbit (H+L)-peroxidase conjugated secondary antibody (Jackson Laboratory, Bar Harbor, ME, USA) for 1 h at 22° C. Protein bands were visualized using an electrogenerated chemiluminescence ECL (Thermo Fisher Scientific, Waltham, MA) bioluminescence method, and images were obtained using a Tanon image system (Tanon, Beijing, China).

### Antioxidant assay

Briefly, approximately 0.25 mg of skin, muscle, kidney, liver, spleen, lung, heart, and brain tissues were transferred into 1.5 mL tubes containing 200 μL of PBS and two steel balls, and the tubes were vortexed in an ice box for 3 min at 65 HZ, followed by centrifugation at 12000 *g* for 15 min. The supernatant was carefully transferred into a 96-well plate without disturbing the precipitate by adding 2;2’-azino-bis (3-ethylbenzthiazoline-6-sulfonic acid) (ABTS; Beyotime, China). The OD values at 450 nm were detected using a microplate reader (BioTek, Winooski, VT, USA).

### Cell proliferation assay

HUVEC were seeded in 96-well plates at a density of 4,000 cells/well and cultured for 24 h. Cell culture media were removed, and the cells were treated with H_2_O_2_, iPSC-derived exosomes, and serum exosomes from iPSC-exosome-treated and untreated mice under different conditions for 24, 48, and 72 h. Cell viability was determined using the Cell Counting Kit-8 (CK04; Dojindo Molecular Technologies, Inc., Kyushu, Japan), and the absorbance was measured at 450 nm using a microplate reader. The assays were performed using six replicates and each experiment was independently repeated five times.

### Measurement of capillary-like structure

HUVEC-GFP were seeded in 24-well plates (1 × 10^4^ cells/well) for 24 h and treated with H_2_O_2_, iPSC-derived exosomes, and serum exosomes from iPSC-exosome-treated and untreated mice for an additional 48 h. Thereafter, HUVEC were digested and seeded into 48-well plates (1 × 10^5^ cells/well) that had been pre-coated with 150 μL of Matrigel (10 mg/mL; BD Biosciences, Franklin Lakes, NJ, USA). After incubation for 12 h, capillary-like structures formed by the endothelial cells were observed using an epifluorescence microscope (BX51; Olympus, Tokyo, Japan). Dil-labeled acetylated low-density lipoprotein (Dil-Ac-LD) were used to measured LDL uptake. The fluorescence intensities of Dil were calculated using Image J software.

### LC-MS/MS and bioinformatics

Serum-derived exosome was collected from old mice, old mice pre-treated with iPSC-derived exosomes via tail vein injection, young mice and young mice pre-treated with iPSC-derived exosomes via tail vein injection. Exosomal protein profiling was performed using LC-MS/MS. MS/MS data were analyzed using Mascot (Matrix Science, London, UK; version 2.3) to search against database. The Search parameters were set as previously described. GO functional annotation of differentially expressed proteins between groups was performed using OmicsBean (http://www.omicsbean.com:88/). Gene expression clusters were correlated with biological process categories, identifying statistically over- and under-represented biological processes among the genes in each cluster by binomial test.

### Data analysis

All the data were analyzed using STATA (StataCorp LLC, College Station, TX, USA) and Prism GraphPad 5.0 software. Significant difference among groups was determined using ANOVA, interaction effect was determined using factorial analysis, and repeated sampling of two samples was analyzed using mixed model analysis. Mean values were considered statistically significant at *p* < 0.05.

## Supplementary Material

Supplementary Figure 1
