# Supplementary material for: iPSC-derived exosomes promote angiogenesis in naturally aged mice
Source: Aging (Albany NY). 2023 Jun 26;15(12):5854–72. doi: 10.18632/aging.204845 (PMC10333073; doi:10.18632/aging.204845)
Supplement: Supplementary Figure 1 [file aging-15-204845-s001.pdf]

SUPPLEMENTARY FIGURE

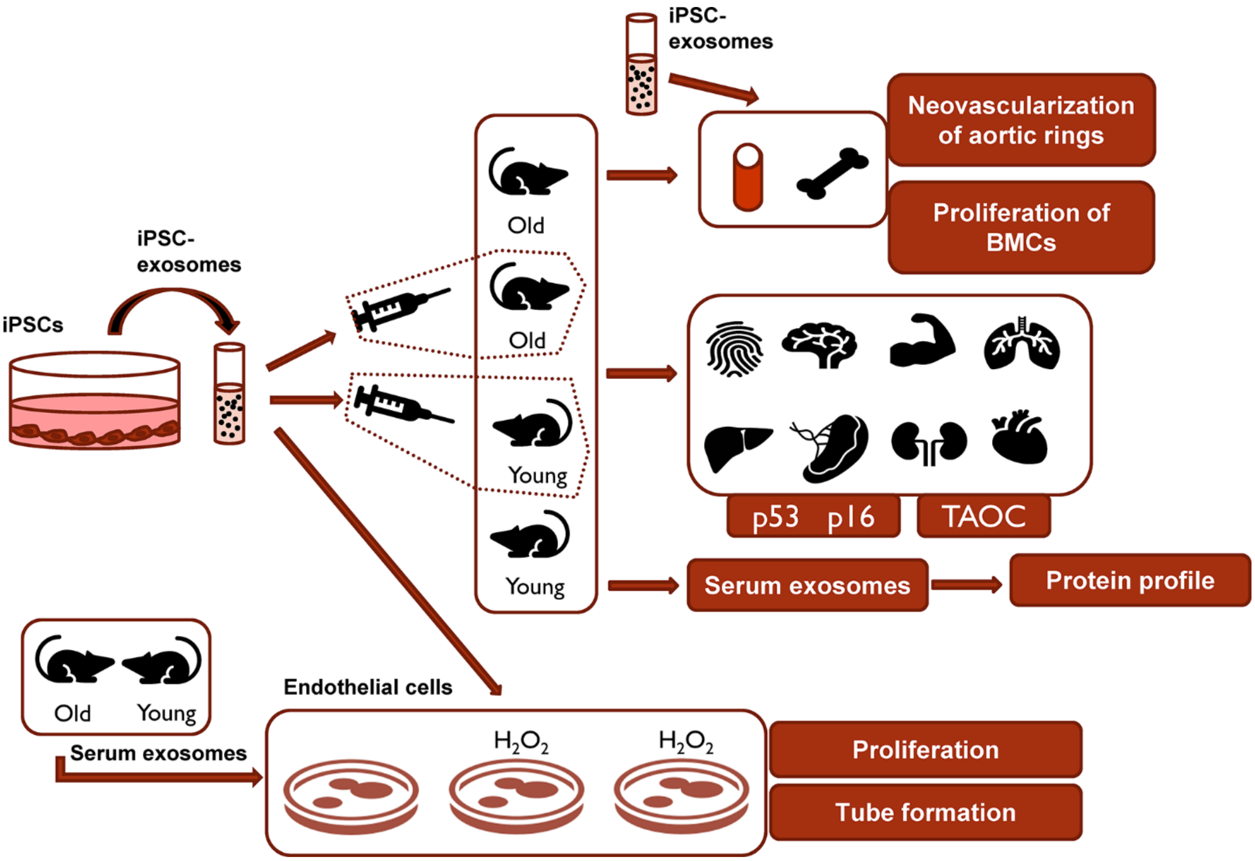

Supplementary Figure 1. Experimental approach. Both *in vitro* culture and *in vivo* pre-injection of iPSC-derived exosomes can restore dysfunctional endothelium. iPSC-derived exosomes can promote angiogenesis, promote the proliferation of BMCs, reduce the expression of p53 and p16, and improve tissue antioxidant capacity in some organs of aging mice. Moreover, iPSC-derived exosomes can repair damaged endothelial function, and improve endothelial cell angiogenesis.
